# CONCEPTIONS OF SUCCESSFUL AGING AMONG OLDER MEN AND WOMEN LIVING WITH HIV

**DOI:** 10.1093/geroni/igac059.1043

**Published:** 2022-12-20

**Authors:** Anna Rubtsova, Tonya Taylor, Marcia Holstad, Gina Wingood, Ighovwerha Ofotokun, Deborah Gustafson, Dilip Jeste, David Moore

**Affiliations:** Emory University, Atlanta, Georgia, United States; SUNY Downstate Medical Center, Brooklyn, New York, United States; Emory University Nell Hodgson Woodruff School of Nursing, Atlanta, Georgia, United States; Columbia University Mailman School of Public Health, New York, New York, United States; Emory University School of Medicine, Atlanta, Georgia, United States; SUNY Downstate, Brooklyn, New York, United States; University of California San Diego, La Jolla, California, United States; University of California, San Diego, La Jolla, California, United States

## Abstract

The aim of this qualitative study was to compare conceptions of successful aging (SA) among older men and women living with HIV. Participants were recruited through 1) Women’s Interagency HIV Study (Atlanta and Brooklyn sites); and 2) HIV Neurobehavioral Research Program at the University of California, San Diego. Our sample included 31 participants: 17 women living with HIV (WLH) and 14 men living with HIV (MLH), age range 51 to 73, 58% Black. We conducted semi-structured interviews in 2018-2021, using the same interview guide for MLH and WLH. The interviews lasted ~90 minutes and were fully transcribed. The thematic and comparative analysis was conducted within the social constructivist paradigm, using MAXQDA software. Although there was some overlap in SA conceptions between MLH and WLH, we found important differences. Whereas maintaining function and general health was more emphasized by MLH, WLH stressed accepting and celebrating aging within the life course perspective.

